# Neonatal resuscitation skills retention among healthcare providers one year after implementation of in situ low-dose high-frequency simulations using innovative tools across two regions in Tanzania

**DOI:** 10.1080/16549716.2026.2639774

**Published:** 2026-03-04

**Authors:** Florence Salvatory Kalabamu, Vickfarajaeli Daudi, Robert Moshiro, Benjamin Kamala, Paschal Mdoe, Dunstan Bishanga, Jan Terje Kvaløy, Hege Ersdal, Rose Mpembeni

**Affiliations:** a School of Public Health and Social Sciences, Muhimbili University of Health and Allied Sciences, Dar es Salaam, Tanzania; b Department of Pediatrics and Child Health, Kairuki University, Dar es Salaam, Tanzania; c Haydom Global Health Research Centre, Haydom Lutheran Hospital, Haydom, Tanzania; d Department of Pediatrics and Child Health, Muhimbili National Hospital, Dar es Salaam, Tanzania; e Directorate of Science, Ifakara Health Institute, Dar es Salaam, Tanzania; f Department of Mathematics and Physics, University of Stavanger, Stavanger, Norway; g Department of Research, Stavanger University Hospital, Stavanger, Norway; h Department of Simulation-Based Learning, Stavanger University Hospital, Stavanger, Norway; i Faculty of Health Sciences, University of Stavanger, Stavanger, Norway

**Keywords:** Safer Births Bundle of Care, NeoNatalie Live, simulation, Helping Babies Breathe, skills training

## Abstract

**Background:**

The Safer Births Bundle of Care (SBBC) was introduced in five regions in Tanzania to improve the quality of care around birth. Among the interventions was an initiative to train healthcare providers on neonatal resuscitation following the Helping Babies Breathe curriculum, using innovative simulation tools.

**Objectives:**

This study aimed to assess simulated neonatal resuscitation skills retention and associated factors one year after implementation.

**Methods:**

A longitudinal study among healthcare providers working in the labour wards of 12 facilities in two regions undergoing SBBC implementation. Healthcare providers were trained and evaluated at baseline and one year after implementation of in situ self-regulated individual skill training. A paired t-test was used to compare the mean difference in skills scores, while factors associated with skills retention were assessed using a modified Poisson regression analysis.

**Results:**

Among 226 participants, 174 (77%) retained skills one year after the implementation. The mean skills score at baseline was 92.5% compared to 82.3% after one year, with a mean difference of 10.2 (95% CI: 8.7–11.7); *p* < 0.001. Sex was independently associated with skills retention; males were less likely to retain skills than females, with an adjusted prevalence ratio (APR) = 0.90 (95%CI: 0.86–0.97); *p* = 0.02.

**Conclusion:**

The majority of healthcare providers retained simulated neonatal resuscitation skills. There was an overall mean skills drop compared to baseline, highlighting the need to strengthen the approaches supporting long-term skills retention. Barriers to and facilitators of sustained effects and scalability should be explored.

## Paper Context

- **Main findings:** The majority of healthcare providers retained newborn resuscitation skills one year after implementation of frequent in-facility self-regulated simulation training using innovative tools. Male sex was significantly associated with skills decay.
- **Added knowledge:** This study highlights the effect of frequent in situ self-regulated simulations using innovative tools to retain neonatal resuscitation skills among healthcare providers in resource-limited settings. This has the potential to improve the quality of care and improved survival of newborns.
- **Global health impact for policy and action:** Incorporation of frequent in situ simulations using innovative training tools as part of continuous quality improvement efforts is important to ensure sustained skills among healthcare providers, with the potential to improve the quality of newborn care.

## Background

It is estimated that around 2.5 million neonates die annually within the first 28 days of birth, with lower- and middle-income countries contributing around 95% of these deaths [1]. In Tanzania, the current neonatal mortality rate is estimated at 24 deaths per 1000 live births [2], which has been stagnant for the past five years [2]. This figure is slightly higher than in the neighbouring countries of Uganda and Kenya, with an estimated 22 deaths per 1000 live births in each country [3,4]. The causes of these deaths, such as birth asphyxia, complications of prematurity and sepsis, are easily preventable through the implementation and scaling-up of interventions aimed to improve quality care around the time of birth [5].

Adequate newborn resuscitation skills among healthcare providers are essential to provide evidence-based quality of care and prevent intrapartum-related complications such as birth asphyxia, which carries a high risk of newborn death [6,7]. Basic newborn resuscitation involves physical stimulation, drying, and ventilation using a bag and mask within one minute (the golden minute) after birth for those babies who fail to establish spontaneous breathing [8]. However, there is evidence of inadequate skills among providers, and some procedures are not done promptly or are implemented incorrectly, leading to undesired outcomes [9]. Moreover, even when initially trained, providers’ skills tend to decay over time as a result of limited refresher training opportunities, heavy workloads, and limited training resources [10]. For instance, a study conducted in Tanzania after a three-year countrywide Helping Babies Breathe (HBB) implementation demonstrated a drop in the percentage of healthcare providers with adequate skills from 87% after baseline training to 56% six months after implementation [11]. This is a potential stumbling block to quality care for newborns, with a subsequent contribution to mortality and morbidity.

In response to the need to scale up innovations with the potential to reduce neonatal mortality, the Safer Births Bundle of Care (SBBC) was implemented in five regions in Tanzania with a large number of deliveries and a high burden of maternal and neonatal deaths [12]. One of the components of the SBBC programme included the training of healthcare providers on the basic resuscitation of neonates using a bag and mask, guided by the Helping Babies Breathe guidelines [8]. To enhance skills acquisition and retention, frequent self-regulated in situ training sessions were introduced using innovative simulation tools [12].

An evaluation of skills after the initial baseline training demonstrated that healthcare providers acquired skills similarly regardless of the differences in their basic characteristics, such as workplace, training and work experience [13]. Furthermore, endline evaluations of the SBBC implementation demonstrated a substantial reduction in 24-hour neonatal deaths by almost 40% [14].

In this study, we aimed to assess simulated neonatal resuscitation skills retention and associated factors one year after implementation of frequent in situ training in selected facilities under SBBC intervention. The findings from this study may help to explain how the SBBC programme reduced neonatal deaths and its potential scalability.

## Methods

### Study design, site, and population

This was a longitudinal study conducted in 12 Comprehensive Emergency Obstetric and Newborn Care health facilities under SBBC implementation in the Geita and Shinyanga regions. These facilities in the respective regions were purposefully selected based on high volumes of births, the higher burden of maternal and newborn mortality, and the absence of other ongoing similar interventions. The study population comprised healthcare providers working in the labour ward and obstetric theatres in the selected facilities. These were doctors and midwives with different levels of training. In the Tanzanian context, certified nurses have two years of formal training; registered nurses have either three years of formal training with a diploma in nursing or four years of formal training with a bachelor’s degree from a university. According to the Tanzanian nursing and midwifery curriculum, both are trained on essential competencies for midwifery practice according to the International Confederation for Midwives guidelines [15].

### Training cascade under the SBBC intervention

#### Training of national facilitators

National SBBC facilitators were selected from active members of professional associations, namely, the Pediatric Association of Tanzania, the Tanzania Nursing and Midwifery Association, and the Association of Gynecologists and Obstetricians in Tanzania. They were selected from the regions where SBBC was being implemented based on their experience working in the labour ward and obstetric theatre, and had prior experience in conducting supportive supervision in maternal and newborn care services. Experts from the SAFER simulation centre trained national facilitators on SimBegin® and European Union Sim-1 courses [16], focusing on how to conduct simulation training, the use and maintenance of SBBC simulation tools and skills evaluation.

#### Training of facility champions

Facility champions (2–3 from each site) were selected from all the SBBC facilities. These were also selected based on their experience in the labour ward and obstetric theatres, and their ability to lead and motivate peers. National facilitators trained the facility champions for six days on how to conduct simulations: their use, troubleshooting and maintenance of training and clinical tools under SBBC. The main roles of the champions were to support national facilitators in conducting in situ training sessions and continuously lead and motivate healthcare providers in their respective facilities to participate in simulation practice.

#### Baseline in-facility training of healthcare providers

**Figure 1.**
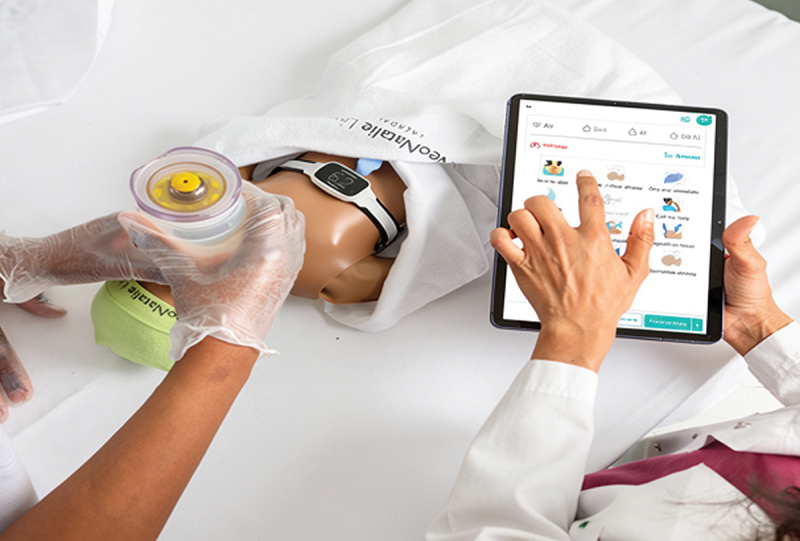
Improved tools for clinical and neonatal resuscitation training (Photos by Laerdal Global Health).

Baseline in-facility training on neonatal resuscitation was conducted in the Geita and Shinyanga regions in November 2021 and January 2022, respectively. These one-day in-facility training sessions were facilitated by national facilitators and facility champions, focusing on simulated neonatal resuscitation of non-breathing neonates using the Helping Babies Breathe curriculum, second version and the Safer Births innovative tools (Figure 1). Participants were given time to practise neonatal resuscitation simulations in groups, switching roles and conducting debriefing after simulations. Furthermore, they were trained in how to maintain and troubleshoot training tools.

##### NeoNatalie live (Laerdal Global Health)

This is a novel newborn simulator which can be connected to a tablet, computer, or smartphone by Bluetooth, whereby learners receive feedback on their training skills performance during neonatal resuscitation practice using a bag and mask resuscitator. The feedback helps the learner to improve their practice over consecutive training sessions [17].

##### The upright resuscitator (Laerdal Global Health)

The mask is designed for effective sealing during bag and mask ventilation practices and has improved ergonomics for effective ventilation [18].

##### NeoBeat (Laerdal Global Health)

This is a neonatal heart rate meter, using dry electrode electrocardiographic technology. It can be attached to a newborn simulator for continuous display of heart rate during ventilation practices. This guides learners in improving their ventilation skills.

#### In situ low-dose high-frequency simulation training

After baseline training, learning corners were established within the labour ward of each site for healthcare providers to practise neonatal resuscitation individually or in team simulations. Facility champions were responsible for coordinating and motivating peers in subsequent in-facility simulation at their own pace, depending on the availability of time and specific skills needs. Therefore, training was self-regulated and motivated by mentorship from national trainers and facility champions. Feedback on practice on NeoNatalie Live was automatically saved in the NeoNatalie Live database, which was used to track the frequency of training and improvement in the quality of training. National facilitators conducted quarterly mentorship and supportive visits to the facilities to support and monitor the progress of training.

### Skills evaluation and data collection

After baseline training, participants were evaluated on their skills in performing neonatal resuscitation using the Objectively Structured Clinical Evaluation (OSCE) of the HBB curriculum, second version (8). Participants who scored more than or equal to 75% were regarded as having passed the skills evaluation as per HBB guidelines. Participants who did not pass the evaluation on their first attempt were given opportunities to practise and repeat the evaluation until they had mastered the skills and passed the evaluation. Findings on skills acquisition and associated factors have been described in a previous publication [13].

The national facilitators performed skills evaluations using an electronic version of the OSCE tool in the Open Data Kit. The tool also captured participants’ basic information such as workplace (region and facility name), facility level, age, level of education, sex, work experience, cadre, and years of experience working in the labour ward. After scoring, evaluation outcomes were automatically saved in a secure central database at Haydom Lutheran Hospital.

At baseline, 481 healthcare providers participated in the initial training and skills evaluation. One year after the implementation of low-dose high-frequency simulation-based training (LDHF-SBT), healthcare providers’ skills were re-evaluated using the same procedure after being notified a week before the activity that the research team would visit the facility and requested all healthcare providers working in the labour ward to be present on a selected day. However, to avoid just-in-time practice from the notification effect, they were not told specifically that it would be a day for skills evaluation. The second evaluation followed those who had participated in the baseline training and continued working in the same facility for one full year. This involved 226/481 healthcare providers. The remaining 255 had shifted to other departments or relocated to other facilities and were not included. Participants who scored ≥75% in skills evaluation after one year of implementation were considered to have retained the skills.

### Data analysis

Data were summarised with means and standard deviations or medians and interquartile ranges for continuous variables and frequency with respective percentages for categorical variables. A comparison of the mean difference in skills scores at baseline and one year after implementation of LDHF-SBT was done using a paired t-test.

To assess factors associated with skills retention, a modified Poisson regression analysis was conducted, as there is a large proportion of positive outcomes. This was done in two steps: First, univariable analysis was conducted, and respective crude prevalence ratios (CPR) with 95% confidence intervals were computed. Second, factors with a *p*-value of less than or equal to 0.2 in univariable analysis were included together in the multivariable model; adjusted prevalence ratios (APR) were computed. Factors with a *p*-value of less than or equal to 0.05 in the second model were considered to be independently associated with skills retention one year after implementation of LDHF-SBT.

Factors associated with neonatal resuscitation skills scores one year after implementation of LDHF-SBT were analysed by a random intercept linear mixed effects model allowing for dependencies within facilities. This approach was selected because data are likely to be correlated within facilities (hospitals). The region was considered as another level; however, since there were only two regions, dependencies within regions were considered by an ordinary fixed effect approach.

First, univariable analysis was conducted by modelling the effect of each explanatory variable on its own while adjusting for dependency within facilities. Next, multivariable analysis was conducted, including all variables together while adjusting for dependency within facilities. Variables with a *p*-value of less than or equal to 0.05 in the final model were considered to be independently associated with neonatal resuscitation skills scores one year after implementation of in situ simulation practices

Data analyses were done using Statistical Package for Social Sciences version 23 (IBM Inc., Chicago, USA) and R version 4.3.3 [19].

### Ethical considerations

This study was conducted in accordance with the Declaration of Helsinki. Moreover, it was approved by the Tanzania National Institute of Medical Research (NIMR) with ethical approval number NIMR/HQ/R.8a/Vol.IX/3458 and the Institutional Review Board of Muhimbili University of Health and Allied Sciences, with approval number MUHAS-REC-03–2023–1603. Participants provided written informed consent before enrolment in the study.

## Results

A total of 226 participants were included in the final analysis. Of these, 200 (88.5%) were nurses while the rest were doctors. The distribution of other sociodemographic characteristics of study participants is shown in Table 1

**Table 1.** Basic demographic characteristics of study participants who completed follow-up (N = 226).

Characteristics		Number	Percentage
Region	Geita	127	56.2
	Shinyanga	99	43.8
Facility level	Health Centre	84	37.2
	District Hospital	70	31.0
	Regional Referral Hospital	72	31.9
Work experience	Less than 1 year	7	3.1
	1–5 years	72	31.9
	More than 5 years	147	65.0
Experience of working in a labour ward	Less than 1 year	17	7.5
	1–5 years	116	51.3
	More than 5 years	93	41.2
Sex	Male	77	34.1
	Female	149	65.9
Age (years)	Under 30	63	27.9
	31–40	119	52.7
	Over 40	44	19.5
Cadre	Nurses	200	88.5
	Doctors	26	11.5
Level of Education*	Certificate	90	39.8
	Diploma	120	53.1
	Degree	16	7.1

*Certificate = two years of formal training; diploma = three years of formal training; degree = four years or more of formal training with a bachelor’s degree from a university.

The attrition rate among participants was comparable in different categories. Higher attrition was observed in those with work experience of less than a year (74.1%); with less than a year’s experience of working in a labour ward (84%); those working in district hospitals (66%), and those with a university degree (66%). Moreover, baseline mean skills scores across and within groups were similar among those who completed and those who were lost to follow-up completion (Supplemental Table S1).

After one year of implementation, the overall median frequency of training among participants was 16, with an interquartile range (IQR) between 5 and 40. Comparison of the median frequency of training among different participant categories is shown in Supplemental Table S2.

Of all participants who completed follow-up, 174 (77%) retained skills one year after the implementation of LDHF-SBT, while 52 (23%) did not retain their skills. The mean skills score dropped from 92.5% at baseline to 82.3% one year after implementation, with a mean difference of 10.2 (95% CI: 8.7–11.7; *p* < 0.001). There was significant skills decay among various participant categories, except for those with work experience of less than a year (*p* = 0.35) and borderline skills decay among those aged under 30 years and between 31 and 40 years (*p* = 0.16 and 0.06), respectively. The mean scores across participants’ characteristics at baseline and one year after implementation of LDHF-SBT are summarised in Table 2.

**Table 2.** Skills scores at baseline and one year after LDHF-SBT implementation across different participant characteristics.

	Scores at baseline	Scores one year after LDHF-SBT		
Characteristics	Mean score (SD)	Mean score (SD)	Mean difference (95% CI)	*P*-value*
**Region**				
Geita	93.0(5.4)	80.4(10.1)	12.7(10.5–14.8)	<0.001
Shinyanga	91.8(5.6)	84.8(9.9)	7.0(4.9–9.0)	<0.001
**Facility level**				
Health Centre	91.0 (5.5)	81.0 (9.5)	10.0(7.6–12.2)	<0.001
District Hospital	94.6(4)	83.0 (11.4)	12.0(8.7–14.7)	<0.001
Regional Referral Hospital	92.3 (6)	83.3(10)	9.0(6.3–11.8)	<0.001
**Work experience**				
Less than a year	84.9 (7)	82(9.2)	3.0(4.3–10.5)	0.35
1–5 years	92.5(6)	82.7(9)	9.8(7.0–12.0)	<0.001
More than 5 years	93.0(4.6)	82(11)	10.6(8.7–12,6)	<0.001
**Experience of working in a labour ward**				
Less than 1 year	91.0(6.3)	88.0(7.8)	3.6(0.07–7.3)	0.05
1–5 years	93.0(5.8)	83.0(9.6)	10.0(8.0–12.0)	<0.001
More than 5 years	92.0(5)	81.0(11)	11.0(8.6–13.8)	<0.001
**Sex**				
Male	93.0(6)	79.0(10.7)	14.0(11.3–17.0)	<0.001
Female	92.3(5)	84.0(9.5)	8.0(6.4–10.0)	<0.001
**Age (years)**				
Under 30	91.0(13)	76.7(5.5)	13.9(14.4–42.0)	0.16
31–40	90.0(8.9)	82.0(11)	8.3(0.3–16.8)	0.06
Over 40	88.8(9)	80.0(10.7)	9.0(3.6–14.7)	<0.01
**Cadre**				
Nurses	92.5(5.5)	82.7(10)	9.8(8.2–11.4)	<0.001
Doctors	92.0(6)	79.0(11.4)	12.8(8.5–17.2)	<0.001
Level of education**				
Certificate	92.0(5.3)	83.0(9.5)	9.5(7.0–11.9)	<0.001
Diploma	91.0(5.5)	81.9(11)	11(8.8–13)	<0.001
Degree	92.0(7.2)	84(9.2)	8.3(3.9–12.7)	0.001
**Overall**	92.5 (6)	82.3(10)	10.2(8.7–11.7)	<0.001

SD = Standard deviation; CI = confidence Interval; *done by paired t-test.

**Certificate = two years of formal training; diploma = three years of formal training; degree = four years or more of formal training with a bachelor’s degree from a university.

### Factors associated with neonatal resuscitation skills retention one year after implementation of LDHF-SBT

In univariable analysis, sex, age of participants, years of experience working in the labour ward and level of education met the criteria for inclusion in a multivariable analysis. In the multivariable model, only sex was independently associated with skills retention; males had lower chances of retaining skills than females (APR = 0.90 (0.86–0.97); *p* = 0.02), as shown in Table 3.

**Table 3.** Factors associated with neonatal resuscitation skills retention one year after implementation of LDHF-SBT (N = 226).

Variables	N (%) who retained skills one year after implementation	CPR (95% CI)	*P*-value	*APR (95% CI)	*P*-value
**Health facility level**					
Health Centre	66(78.6)	1.0(0.9–1.1)	0.75		
District Hospital	53(75.7)	1.0(0.9–1.1)	0.93		
Regional Referral Hospital	55(76.4)	Ref.			
**Regions**					
Geita	96(75.6)	0.98(0.9–1)	0.57		
Shinyanga	78(78.8)	Ref.			
**Work experience (years)**					
Less than 1 year	5(71.4)	0.9(0.8–1)	0.74		
1–5 years	55(77.8)	1.0(0.9–1.1)	0.88		
More than 5 years	113(76.9)	Ref.			
**Age (years)**					
Under 30	52(82.5)	1.0(0.9–1.1)	0.52	1(0.9–1.1)	0.82
31–40	88(93.9)	1.0(0.9–1.1)	0.65	0.9(0.8–1.1)	0.48
Over 40	34(77.3)	Ref.			
**Experience of working in a labour ward**					
Less than 1 year	15(88.2)	1.1(1–1.2)	0.13	1(0.9–1.2)	0.43
1–5 years	92(79.2)	1.0(0.9–1.1)	0.21	1(0.9–1.1)	0.42
More than 5 years	67(72)	Ref.			
**Sex**					
Male	53(68.8)	0.90(0.87–0.98)	0.03	0.90(0.86–0.97)	0.02
Female	121(81.2)	Ref.			
**Level of education**					
Certificate	68(75.6)	0.9(0.8–1)	0.1	0.9(0.8–1)	0.1
Diploma	91(75.8)	0.9(0.8–1)	0.1	0.9(0.8–1)	0.13
Degree and above	15(93.8)	Ref.			
**Cadre of the** Healthcare Providers s					
Nurses	152(76.8)	0.99(0.9–1)	0.83		
Doctors	22(78.6)	Ref.			
**Average individual training sessions per month**					
F than 4	137(76.1)	0.9(0.7–1.3)	0.84		
More than or equal to 4	37(80.4)	Ref.			

*Adjusted Prevalence Ratio is the measure used to compare the probability of the outcome of interest (skills retention) adjusted for other confounders. Used when the outcome of interest is common.

### Factors associated with skills scores one year after the implementation of in situ LDHF-SBT

In univariable analysis, age was negatively correlated with skills scores, showing that scores decreased by 0.16 for each year of increase in age (*p* = 0.04). Moreover, experience working in a labour ward had a significant effect on skills; those in the more than 5 years group scored 2.7 points lower than the 1–5 years group (*p* = 0.05). The overall effect is that the more experience, the lower the skills scores. Similarly, males had significantly lower scores than females (*p* < 0.001). However, on multivariable analysis with all explanatory variables included in the model, only sex was independently associated with skill scores one year after implementation of in situ simulations. Males scored significantly lower than females (*p* = 0.001), as shown in Supplemental Table S3.

## Discussion

In this study, we aimed to assess simulated neonatal resuscitation skills retention one year after implementation of LDHF-SBT using innovative tools in 12 facilities under SBBC implementation. We found that the majority of study participants (77%) were able to retain skills above the threshold. However, there was a significant drop in overall mean skills scores compared to immediately after baseline training. Furthermore, we found that sex was independently associated with skills retention and skills scores one year after implementation of in situ simulations, in which females retained skills significantly and had better scores than males one year after implementation.

The observed skills fall-off in this study is low compared to other similar studies in similar settings. For instance, results from a large-scale implementation of HBB in Tanzania demonstrated a decrease in the percentage of healthcare providers who passed skills evaluation by 31.3% after six months of implementation compared to a 23% drop observed in this study after one year of implementation [11]. The trend is similar to other studies in Tanzania and elsewhere, showing a skills drop, especially on mastery of bag and mask ventilation [20–23].

In contrast to what was expected in this study, participants with more working experience had more skills decay than juniors with less work experience, even though the median frequency of training was not significantly different. There is no clear explanation for this finding; however, it could be linked to overconfidence with little attention to following the steps in the resuscitation algorithm.

In this study, the majority of participants retained skills, which supports similar studies reporting that the use of frequent simulation training ensures consistency in skills with the potential to improve the quality of neonatal care and a reduction in mortality [24,25]. This is important because neonatal resuscitation with a bag and mask is not a routine event, as only 5–10% of neonates need bag and mask resuscitation; therefore, it is essential for healthcare providers to practise more on developing these skills using frequent simulations.

In our recent publication on the impact of SBBC, we demonstrated a risk reduction in 24-hour neonatal deaths by almost 40% [14]. This achievement could be linked to the current study’s findings, demonstrating that frequent in situ simulation training has the potential to impart skills among healthcare providers with a subsequent improvement in the quality of neonatal care. In this study, healthcare providers conducted frequent simulations at the learning corners in the labour ward – the environment where they usually perform their daily routine duties – followed by team debriefing. This has been shown to enhance knowledge sharing, skills development and retention, teamwork, communication, and increased confidence among themselves to manage the most challenging clinical incidents [26,27]. These findings are supported by other similar studies conducted in Ghana and Uganda, which linked frequent simulation with skills retention and subsequent improvement in clinical practice and a reduction in 24-hour neonatal mortality [22,24].

Furthermore, the use of facility champions for coordination and encouraging peers to conduct simulations, and frequent supervision visits conducted by SBBC national trainers during the implementation period could have contributed to most healthcare providers retaining their skills over the one-year review period. This is also supported by Vadla et al. in a similar setting where the use of in-facility training motivators improved the frequency of practice and skills [28].

Generally, females retained skills than males. This difference is not well understood, and previous studies on sex differences in medical simulation performance are inconclusive, indicating that the outcome depends on the task involved and the context [29]. However, historically, globally and in local contexts, nursing and midwifery fields have been dominated by females, which could also motivate females to train with more focus and intention, with better subsequent skills mastery and retention than males. Therefore, the frequency of training alone could not explain the better performance among females, as the median frequency of training was similar in both sexes. This suggests that internal motivation for skills mastery among females could be contributing to more focus and concentration during training sessions.

### Limitations

This study faced limitations; first, this study was part of a quality improvement project with little control over routine health systems’ functions and plans. During the study period, half of the healthcare providers who were enrolled in the study at its inception had either relocated to other facilities and departments or had gone on to further studies. Similarly, other newly employed healthcare providers started working in the departments during the implementation phase. This could affect the final results; however, this was mitigated by including only those who were present during the initial training and evaluation at the start of the study and continued to participate in frequent in situ simulations in the same facilities. In Tanzania, there is a practice of task sharing where healthcare providers with some skills can be shifted to other sections in need of those skills as a result of the inadequacy of human resources for health; however, efforts are ongoing to train more specialised midwives who will work exclusively in labour wards. Possibly, this will ensure the retention of well-trained and experienced birth attendants in the labour wards. The strength of this study is underpinned by the utilisation of facility champions to plan and coordinate training with minimal influence from the research team.

## Conclusion

In conclusion, the majority of healthcare workers retained skills one year after the implementation of in situ self-regulated simulation skills training for neonatal resuscitation using innovative tools. This suggests that frequent self-regulated training, motivated by well-trained and motivated in-facility champions using improved technological tools, is possible in low-resource settings, and can be easily integrated into the existing health system as part of continuous quality improvement. To ensure sustainability and maximise its potential, barriers and facilitators of frequent in situ simulation training should be well elucidated to guide continuous improvement efforts and the scalability of the SBBC programme. Furthermore, factors identified as affecting the retention of skills should be further studied in large-scale and multi-centre studies.

## Supplementary Material

STROBE_checklist.doc

Supplemental materials_18Feb2026.docx

## Data Availability

Data can be made available upon written request to the Institutional Review Board of the Muhimbili University of Health and Allied Sciences and the Tanzania National Institute of Medical Research.

## References

[1] Rosa-Mangeret F, Caroline BA, Golaz A, et al. 2.5 million annual deaths—are neonates in low- and middle-income countries too small to be seen? A bottom-up overview on neonatal morbi-mortality. Trop Med Infect Dis. 2022;7:64. https://www.mdpi.com/2414-6366/7/5/64 35622691 10.3390/tropicalmed7050064 PMC9148074

[2] NBS. Tanzania Demographic and Health Survey and Malaria Indicator Survey 2022 key indicators. Dodoma: NBS; 2022.

[3] Mwangi A, Yego F. Neonatal mortality estimates and associated risk factors in nine counties in Kenya. North Carolina: University of Carolina; 2023.

[4] Uganda Ministry of Health. Situation analysis of newborn health in Uganda_2023 update. Kampala: MoH; 2023.

[5] Mangu CD, Rumisha SF, Lyimo EP, et al. Trends, patterns and cause-specific neonatal mortality in Tanzania: a hospital-based retrospective survey. Int Health. 2021;13:334–9. https://academic.oup.com/inthealth/article/13/4/334/5911609 32975558 10.1093/inthealth/ihaa070 PMC8253992

[6] Msemo G, Massawe A, Mmbando D, et al. Newborn mortality and fresh stillbirth rates in Tanzania after helping babies breathe training. Pediatrics. 2013;131:e353–e360. 23339223 10.1542/peds.2012-1795

[7] Walker D, Otieno P, Butrick E, et al. Effect of a quality improvement package for intrapartum and immediate newborn care on fresh stillbirth and neonatal mortality among preterm and low-birthweight babies in Kenya and Uganda: a cluster-randomised facility-based trial. Lancet Glob Health. 2020;8:e1061–70. doi: 10.1016/S2214-109X(20)30232-1 32710862 PMC7388203

[8] Helping Babies Breath Global Implementation Taskforce. Guide for implementation of helping babies breathe®. 2011 [cited 2026 Feb 13]. Available from: http://www.helpingbabiesbreathe.org/docs/IG_pdfs/Impl.guide 22 desember 2011.pdf

[9] Ersdal HL, Mduma E, Svensen E, et al. Early initiation of basic resuscitation interventions including face mask ventilation may reduce birth asphyxia related mortality in low-income countries. Resuscitation. 2012;83:869–873. doi: 10.1016/j.resuscitation.2011.12.011 22198423

[10] Daka M, Tsarkov A, Petlovanyi P, et al. Self-efficacy and determinants of newborn resuscitation practices among healthcare professionals in Chipata District, Eastern Province, Zambia. Eur J Clin Med. 2024 Jun 19;5:22–31. doi: 10.24018/clinicmed.2024.5.3.341

[11] Arlington L, Kairuki AK, Isangula KG, et al. Implementation of “helping babies breathe”: a 3-year experience in Tanzania. Pediatrics. 2017;139:e20162132. doi: 10.1542/peds.2016-2132 28557724

[12] Kamala BA, Ersdal HL, Mduma E, et al. SaferBirths bundle of care protocol: a stepped-wedge cluster implementation project in 30 public health-facilities in five regions, Tanzania. BMC Health Serv Res. 2021;21:1–13. doi: 10.1186/s12913-021-07145-1 34663296 PMC8524841

[13] Kalabamu FS, Daudi V, Moshiro R, et al. Neonatal resuscitation skills acquisition among healthcare providers after Helping Babies Breathe simulation training using improved tools across two regions in Tanzania. Adv Simul. 2025;10:6. doi: 10.1186/s41077-025-00338-2 PMC12362981 40025598

[14] Kamala BA, Ersdal HL, Moshiro RD, et al. Outcomes of a program to reduce birth-related mortality in Tanzania. N Engl J Med. 2025;392:1100–1110. doi: 10.1056/NEJMoa2406295 40009803

[15] ICM. Essential competencies for midwifery practice. The Hague: ICM; 2024.

[16] Simbegin. A faculty development program designed for new simulation educators [Internet]. Simbegin. [cited 2023 Oct 5]. Available from: https://www.safer.net/simbegin/

[17] Curran V, Fleet L, White S, et al. A randomized controlled study of manikin simulator fidelity on neonatal resuscitation program learning outcomes. Adv Health Sci Educ. 2015;20:205–218. doi: 10.1007/s10459-014-9522-8 24916954

[18] Thallinger M, Ersdal HL, Ombay C, et al. Randomised comparison of two neonatal resuscitation bags in manikin ventilation. Arch Dis Child Fetal Neonatal Ed. 2016;101:F299–303. doi: 10.1136/archdischild-2015-308754 26437670

[19] R Core Team. A language and environment for statistical computing. R Foundation for Statistical Computing. Vienna: R Foundation for Statistical Computing; 2016.

[20] Makene CL, Plotkin M, Currie S, et al. Improvements in newborn care and newborn resuscitation following a quality improvement program at scale: results from a before and after study in Tanzania. BMC Pregnancy Childbirth. 2014;14:1–11. doi: 10.1186/s12884-014-0381-3 25406496 PMC4247559

[21] Reisman J, Arlington L, Jensen L, et al. Newborn resuscitation training in resource-limited settings: a systematic literature review. Pediatrics. 2016;138. doi: 10.1542/peds.2015-4490 27388500

[22] Lynn Evans C, Bazant E, Atukunda I, et al. Peer-assisted learning after onsite, low-dose, high-frequency training and practice on simulators to prevent and treat postpartum hemorrhage and neonatal asphyxia: a pragmatic trial in 12 districts in Uganda. PLoS One. 2018;13:1–17. doi: 10.1371/journal.pone.0207909 PMC6296740 30557350

[23] Ugwa E, Kabue M, Otolorin E, et al. Simulation-based low-dose, high-frequency plus mobile mentoring versus traditional group-based trainings among health workers on day of birth care in Nigeria; a cluster randomized controlled trial. BMC Health Serv Res. 2020;20:586. doi: 10.1186/s12913-020-05450-9 32590979 PMC7318405

[24] Gomez PP, Nelson AR, Asiedu A, et al. Accelerating newborn survival in Ghana through a low-dose, high-frequency health worker training approach: a cluster randomized trial. BMC Pregnancy Childbirth. 2018;18:72. doi: 10.1186/s12884-018-1705-5 29566659 PMC5863807

[25] Evans CL, Bazant E, Atukunda I, et al. Peer-assisted learning after onsite, low-dose, high-frequency training and practice on simulators to prevent and treat postpartum hemorrhage and neonatal asphyxia: a pragmatic trial in 12 districts in Uganda. PLoS One. 2018;13:e0207909. doi: 10.1371/journal.pone.0207909 30557350 PMC6296740

[26] Alanazi A, Nicholson N, Thomas S. The use of simulation training to improve knowledge, skills, and confidence among healthcare students: a systematic review. Internet J Allied Health Sci Pract. 2017;15:2. doi: 10.46743/1540-580X/2017.1666

[27] Ayaz O, Wasim Ismail F. Healthcare simulation: a key to the future of medical education-a review. Adv Med Educ Pract. 2022;13:301–308. doi: 10.2147/AMEP.S353777 35411198 PMC8994530

[28] Vadla MS, Mdoe P, Moshiro R, et al. Neonatal resuscitation skill-training using a new neonatal simulator, facilitated by local motivators: two-year prospective observational study of 9000 trainings. Children [Internet]. 2022;9:134. doi: 10.3390/children9020134 35204855 PMC8870207

[29] Ali A, Subhi Y, Ringsted C, et al. Gender differences in the acquisition of surgical skills: a systematic review. Surg Endosc. 2015;29:3065–3073. doi: 10.1007/s00464-015-4092-2 25631116

