# Supplementary material for: Neonatal resuscitation skills retention among healthcare providers one year after implementation of in situ low-dose high-frequency simulations using innovative tools across two regions in Tanzania
Source: Glob Health Action. 2026 Mar 4;19(1):2639774. doi: 10.1080/16549716.2026.2639774 (PMC12961705; doi:10.1080/16549716.2026.2639774)
Supplement: Supplemental materials_18Feb2026.docx [file ZGHA_A_2639774_SM7093.docx]

**Supplemental table 1: Comparison of characteristics between participants who completed follow up and who were lost to follow up**

| **Characteristics** | | **Completed follow up (N=226)** | | | **Did not complete follow up (N=255)** | | | **Attrition rate (%)** | **ϮP value** |
| --- | --- | --- | --- | --- | --- | --- | --- | --- | --- |
|  |  | **n** | **%** | **Mean skills score at baseline** | **n** | **%** | **Mean skills score at baseline** |  |  |
| **Region** | Geita | 127 | 56.2 | 93.0 | 106 | 41.6 | 92.3 | 45.7 | 0.001 |
|  | Shinyanga | 99 | 43.8 | 91.8 | 149 | 58.4 | 92.8 | 59.5 |  |
|  |  |  |  |  |  |  |  |  |  |
| **Facility level** | Health Center | 84 | 37.2 | 91.0 | 74 | 29.0 | 90.9 | 59.7 | 0.24 |
|  | District Hospital | 70 | 31.0 | 94.6 | 116 | 45.5 | 93.3 | 66.3 |  |
|  | Regional Referral Hospital | 72 | 31.9 | 92.3 | 65 | 25.5 | 93.2 | 48.2 |  |
|  |  |  |  |  |  |  |  |  |  |
| **Work experience** | Less than a year | 7 | 3.1 | 84.9 | 13 | 5.1 | 91.2 | 74.1 | 0.5 |
|  | 1-5 years | 72 | 31.9 | 92.5 | 58 | 22.7 | 93.2 | 39 |  |
|  | More than 5 years | 147 | 65.0 | 93.0 | 184 | 72.2 | 92.5 | 56.3 |  |
|  |  |  |  |  |  |  |  |  |  |
| **Experience working in labour ward** | Less than 1 year | 17 | 7.5 | 91.0 | 13 | 5.1 | 90.8 | 84 | 0.07 |
|  | 1-5 years | 116 | 51.3 | 93.0 | 58 | 22.7 | 93.0 | 53 |  |
|  | More than 5 years | 93 | 41.2 | 92.0 | 184 | 72.2 | 93.1 | 27.3 |  |
|  |  |  |  |  |  |  |  |  |  |
| **Sex** | Male | 77 | 34.1 | 93.0 | 85 | 33.3 | 92.9 | 58.4 | 0.02 |
|  | Female | 149 | 65.9 | 92.3 | 170 | 66.7 | 92.4 | 49.7 |  |
|  |  |  |  |  |  |  |  |  |  |
| **Age (years)** | Less than 30 | 63 | 27.9 | 91.0 | 64 | 25.1 | 92.2 | 53.3 | 0.19 |
|  | 31-40 | 119 | 52.7 | 90.0 | 131 | 51.4 | 92.8 | 51 |  |
|  | Above 40 | 44 | 19.5 | 88.8 | 64 | 25.1 | 92.5 | 57 |  |
|  |  |  |  |  |  |  |  |  |  |
| **Cadre** | Nurses | 200 | 88.5 | 92.5 | 225 | 88.2 | 92.6 | 52.5 | 0.68 |
|  | Doctors | 26 | 11.5 | 92.0 | 30 | 11.8 | 92.2 | 56.7 |  |
|  |  |  |  |  |  |  |  |  |  |
| **Level of Education⃰** | Certificate | 90 | 39.8 | 92.0 | 113 | 44.3 | 92.1 | 54.1 | 0.05 |
|  | Diploma | 120 | 53.1 | 91.0 | 124 | 48.6 | 93.2 | 49.6 |  |
|  | Degree | 16 | 7.1 | 92.0 | 18 | 7.1 | 91.6 | 66 |  |
| *^⃰^Certificate = two years of formal training; diploma = three years of formal training; degree = four years or more of formal training with a bachelor’s degree from universities*  *^Ϯ^= p value from Chisquare test for comparison of frequency within sociodemographic categories* | | | | | | | | | |

**Supplemental table 2: Median frequency of training within one year of implementation of LDHF-SBT for neonatal resuscitation (N=266)**

| **Characteristics** | | **Median Frequency of training**  **(IQR)** | **P value** |
| --- | --- | --- | --- |
| **Region** | Geita | 13 (4-31) | ^⁕^0.06 |
|  | Shinyanga | 28(7-59) |  |
|  |  |  |  |
| **Facility level** | Health Center | 18(8-42) | ⃰0.35 |
|  | District Hospital | 14(4-35) |  |
|  | Regional Referral Hospital | 14(4-35) |  |
|  |  |  |  |
| **Work experience** | Less than a year | 12(7-47) | ⃰0.35 |
|  | 1-5 years | 13(3-33) |  |
|  | More than 5 years | 18(6-41) |  |
|  |  |  |  |
| **Experience working in labour ward** | Less than 1 year | 12(1-39) | ⃰0.18 |
|  | 1-5 years | 23(4-32) |  |
|  | More than 5 years | 20(7-44) |  |
|  |  |  |  |
| **Sex** | Male | 15(4-42) | ^⁕^0.94 |
|  | Female | 16(5-38) |  |
|  |  |  |  |
| **Age (years)** | Less than 30 | 12(3-32) | ⃰0.38 |
|  | 31-40 | 18(6-105) |  |
|  | Above 40 | 15(3-45) |  |
|  |  |  |  |
| **Cadre** | Nurses | 16(6-40) | ^⁕^0.68 |
|  | Doctors | 12(4-40) |  |
|  |  |  |  |
| **Level of Education** | Certificate | 16(6-41) | ⃰0.88 |
|  | Diploma | 16(4-40) |  |
|  | Degree | 12(2-45) |  |
| **⃰***Kruskal-Wallis H Test*  *^⁕^Mann-Whitney U Test*  *IQR=interquartile range*  *LDHF-SBT=Low dose high frequency simulation-based training* | | | |

**Supplemental Table 3: Factors associated with neonatal resuscitation skills scores at the end of one year of LDHF-SBT implementation (N=226).**

| **Variable name** | **Univariable analysis** | | **Multivariable analysis** | |
| --- | --- | --- | --- | --- |
|  | **⃰Effect size (β)**  **(95% CI)** | **P value** | **⃰Effect size (β)**  **(95% CI)** | **P value** |
| **Region** |  |  |  |  |
| Geita (ref.) |  |  |  |  |
| Shinyanga | 1.6(-4.6, 7.9) | 0.58 | 1.5 (-6.1, 9.1) | 0.66 |
| **Facility level** |  |  |  |  |
| District hospital (Ref.) |  |  |  |  |
| Health Center | -1.8(-9.8, 6.2) | 0.62 | -0.64 (-9.7, 8.4) | 0.87 |
| Regional referral hospital | 1.3(-8.8, 11) | 0.78 | 0.73 (-11, 12) | 0.89 |
| **Age (years)** |  |  |  |  |
| Age (years) | -0.16(-0.31,0.01) | 0.04 | -0.07(-0.26, 0.12) | 0.47 |
| **Working experience (years)** |  |  |  |  |
| Between 1-5 years (ref) |  |  |  |  |
| Less than a year | -3.3(-11, 4.3) | 0.4 | -6.9 (-15, 1.4) | 0.1 |
| More than 5 years | -1.9(-4.6, 0.94) | 0.19 | -0.83 (-4.3, 2.6) | 0.63 |
| **Experience working in the labour ward (years**) |  |  |  |  |
| Between 1-5 years (ref) |  |  |  |  |
| Less than 1 year | 3.6(-1.2, 8.5) | 0.14 | 4.8 (-0.88, 11) | 0.1 |
| More than 5 years | -2.7(-5.3, -0.03) | 0.05 | -1.3 (-4.6, 2.0 ) | 0.44 |
| **Sex** |  |  |  |  |
| Female (ref) |  |  |  |  |
| Male | -4.6(-7.2, -1.9) | <0.001 | -4.6 (-7.4, -1.8) | 0.001 |
| **Cadre** |  |  |  |  |
| Doctors (ref) |  |  |  |  |
| Nurse-midwives | 1.8(-2.3, 5.8) | 0.39 | 3.6 (-1.9, 9.0) | 0.2 |
| **Level of education** |  |  |  |  |
| Certificate (ref.) |  |  |  |  |
| Degree | 3.2(-2.0, 8.4) | 0.22 | 6.0 (-1.3, 13) | 0.11 |
| Diploma | 0.2(-2.5, 2.8) | 0.90 | 1.1 (-1.5, 3.8) | 0.4 |
|  |  |  |  |  |
| ⃰*Effect size/coefficient (β) shows the measure of strength and direction (positive or negative) of the relationship between the independent and the dependent (outcome) variable. Represents the change in outcome variable for one unit increase or change from one category to another.*  *CI= Confidence interval* | | | | |
